# Status quo, influencing factors, and association with hospitalization outcomes of malnutrition in neurological disorders in China: a national cross-sectional study

**DOI:** 10.3389/fnut.2025.1633212

**Published:** 2025-11-07

**Authors:** Lan Lan, Shuyan Guo, Yujia Han, Shuo Zhai, Yan Liu, Pengfei Li, Xudong Zhang, Siping Dong

**Affiliations:** 1 Information Management and Data Center, Beijing Tiantan Hospital, Capital Medical University, Beijing, China; 2 National Institute of Hospital Administration, National Health Commission of the People's Republic of China, Beijing, China; 3 Center for Clinical and Epidemiologic Research, Beijing Anzhen Hospital, Capital Medical University, Beijing, China; 4 Department of Neurology, Beijing Tiantan Hospital, Capital Medical University, Beijing, China

**Keywords:** malnutrition, neurological disorders, GLIM, prevalence, influencing factor, hospitalization outcome

## Abstract

**Background:**

In recent years, the issue of malnutrition in people with neurological disorders has emerged as a growing concern. However, there is still a lack of global research on the current status of malnutrition in these disorders in China, which warrants further investigation. This study aims to clarify the prevalence of malnutrition in these disorders, evaluate its contributing factors, and assess its impact on hospitalization outcomes.

**Method:**

Based on the China Nutrition Fundamental Data 2020 (CNFD 2020) Project, this nationwide survey was conducted from February to October 2023 across 31 provincial-level administrative regions of China, utilizing questionnaires for data collection. The study included 1,357 patients with neurological diseases, whose nutritional status was systematically assessed using the standardized Global Leadership Initiative on Malnutrition (GLIM) diagnostic framework. Statistical analyses were performed using descriptive statistics, multivariate logistic regression, random forest, Kruskal-Wallis test and multiple linear regression.

**Results:**

The overall prevalence of malnutrition in patients with neurological disorders was 32.5%, including 30.8% moderate malnutrition and 1.7% severe malnutrition. Multivariate logistic regression identified neutrophil percentage (OR = 1.026, *P* < 0.001, 95%CI: 1.015–1.038) and western region (OR = 1.319, *P* < 0.001, 95%CI: 1.002–1.734) as significant influencing factors for malnutrition. Random forest analysis showed that lymphocyte percentage, red blood cell count and education level ranked highest in SHapley Additive exPlanations (SHAP) values. Boxplot analysis demonstrated that malnutrition status may increase both length of stay and hospitalization costs.

**Conclusions:**

Malnutrition is prevalent in patients with neurological disorders, with neutrophil percentage, lymphocyte percentage, red blood cell count, region, and education level identified as significant contributing factors. Malnutrition may be associated with increased length of stay and hospitalization costs.

## Introduction

Neurological disorders are highly heterogeneous, characterized by complex pathogenic mechanisms and diverse clinical manifestations that severely impair patients' quality of life. These conditions are often associated with cognitive dysfunction, feeding difficulties, and impaired consciousness, which together increase nutritional risk and predispose patients to malnutrition (1). In people with neurological disorders, malnutrition can exacerbate the primary condition, prolong hospitalization, increase healthcare costs, and adversely affect clinical outcomes (2). Therefore, systematic nutritional assessment in these patients is essential to improve prognosis and enhance quality of life.

Recent studies of malnutrition in neurological patients have highlighted the critical influence of nutritional status on rehabilitation and clinical outcomes. Current research has focused on various neurological disorders, including ischemic stroke, Alzheimer's disease, and Parkinson's disease, and has extensively investigated the prevalence of nutritional risk, malnutrition, and their associations with clinical outcomes in China (3–6). A variety of nutritional assessment tools-such as controlling nutritional status (CONUT), mini nutritional assessment short form (MNA-SF), and Global Leadership Initiative on Malnutrition (GLIM)-have been validated in different diseases and populations. Disease-specific nuances in malnutrition patterns are evident. For example, acute ischemic stroke patients at risk of malnutrition on admission show strong correlations with poor prognosis, including prolonged hospitalization, neurological deterioration after stroke, increased risk of infection and mortality. Similarly, malnutrition in Parkinson's disease is strongly associated with both motor and non-motor symptoms, disease severity, and may exacerbate cognitive decline and depressive symptoms (5, 7, 8). Current research focuses on elucidating the mechanistic pathways by which malnutrition affects clinical outcomes, with efforts to develop predictive models using various nutritional assessment tools for personalized intervention strategies.

Malnutrition is prevalent in neurological patients and has a profound impact on clinical prognosis and quality of life (9). While existing studies have tentatively established associations between malnutrition and neurological disorders, significant controversies and limitations remain (10). In terms of data sources, most current research is derived from single-center studies limited to specific regional or institutional populations, which limits generalizability (11, 12). Small sample sizes further limit the detection of subtle effects or differences. Methodologically, despite the widespread clinical use of various dietary assessment tools, their applicability to specific diseases and populations remains controversial. Variations in sensitivity, specificity, and predictive validity of these tools continue to fuel debate about optimal assessment methods (13).

In this study, we analyzed data from hospitals in 31 provinces, municipalities and autonomous regions in China to investigate the prevalence and factors associated with malnutrition risk on admission and its association with hospitalization outcomes in neurological patients.

## Methods

### Study design

Data were obtained from the China Nutrition Fundamental Data 2020 (CNFD 2020) Project (14), which was conducted from February to October 2023 in 31 provinces, municipalities and autonomous regions in China using a multi-stage stratified cluster sampling method based on China's administrative divisions. In the first stage, 31 provinces, municipalities and autonomous regions were selected in China. The second stage involved selecting 1–23 tertiary hospitals and 1–2 secondary hospitals with nutrition departments from each province, with one provincial key hospital designated as the coordinating center. In the third stage, continuous sampling was conducted at each site until the predetermined target sample size (200 patients per tertiary hospital and 150 patients per secondary hospital) was reached.

Participants were included in the survey if they were aged 18 years or over, had been hospitalized for tumors or diseases of the respiratory, digestive, endocrine, cardiovascular, urinary or nervous systems, and had completed the data collection within 24–48 h of admission. In this study, the inclusion criteria were further refined to include only participants with a primary diagnosis of neurological disease from the surveyed population. The exclusion criteria included pediatric and critically ill patients, individuals with mental disorders or memory impairment who could not provide accurate responses, participants who lacked decision-making capacity, and other patients deemed ineligible by the investigators. In this study, those who did not undergo malnutrition risk screening were further excluded.

In terms of data collection, dietitians and clinicians conducted face-to-face interviews with patients to gather sociodemographic data; anthropometric measurements were performed according to standard protocols; laboratory data were extracted from the hospital information system; and body composition was measured using BIA (for some patients).

This study strictly adhered to the guidelines of the STROBE Statement and received ethical approval from the Ethics Committee of Peking Union Medical College Hospital (I-22PJ744), with all participants providing verbal informed consent prior to the survey.

The classification of eastern, central, and western regions in this study follows the standard established by the National Bureau of Statistics of China. This classification groups China's 31 provincial-level administrative regions based on geographic location and socioeconomic development levels. Specifically, the eastern region comprises the economically advanced coastal provinces; the central region serves as a major base for grain production and energy raw materials; while the western region is less developed economically and encompasses predominantly inland and remote areas.

### Variable definitions

#### Malnutrition

Nutritional risk screening was first performed using the Nutritional Risk Screening 2002 (NRS-2002) tool to identify patients at risk of malnutrition, followed by assessment of nutritional status using the GLIM criteria. The GLIM diagnostic framework includes three phenotypic criteria and two etiologic criteria. A diagnosis of malnutrition required the fulfillment of at least one phenotypic and one etiologic criterion. The three phenotypic criteria are ① weight loss: >5% weight loss in the last 6 months or >10% weight loss over 6 months; ② low BMI: BMI < 18.5 kg/m^2^ for patients aged < 70 years and < 20 kg/m^2^ for those ≥70 years; ③ reduced muscle mass: calf circumference < 34 cm for men and < 33 cm for women, or appendicular skeletal muscle mass index (ASMI) < 7.0 kg/m^2^ for men and < 5.7 kg/m^2^ for women, or handgrip strength < 28 kg for men and < 18 kg for women (15–17). The two etiologic criteria are ① reduced food intake or assimilation: energy intake < 50% of requirement for >1 week, or persistently reduced intake for >2 weeks, or chronic gastrointestinal disease affecting nutrient absorption; ② disease burden/inflammation: acute disease/injury with severe inflammation or chronic disease with moderate chronic/recurrent inflammation. Indicators such as high-sensitivity C-reactive protein (>3 mg/L), albumin (< 30 g/L), and body temperature (>38 °C) were used as supportive proxy measures of inflammation (18).

#### Stages of malnutrition

The GLIM criteria use only phenotypic criteria to classify the severity of malnutrition, with the diagnosis of stage II/severe malnutrition requiring at least one phenotypic criterion. In this study, the severity of malnutrition was classified into moderate and severe categories using the following diagnostic thresholds: ① moderate malnutrition was defined as a weight loss of 5%−10% within 6 months and 10%−20% beyond 6 months, whereas severe malnutrition required a weight loss of >10% within 6 months and >20% beyond 6 months; ② moderate ranges were BMIε[17.0 kg/m^2^, 18.5 kg/m^2^) for age < 70 years and BMIε[17.8 kg/m^2^, 20 kg/m^2^) for age ≥70 years, with severe defined as BMI < 17.0 kg/m^2^ for age < 70 years and BMI < 17.8 kg/m^2^ for age ≥70 years (19); ③ moderate thresholds included calf circumference menε[26.3cm, 34 cm) and womenε[25.2 cm, 33 cm) (15, 16), or ASMI menε[5.68 kg/m^2^, 7.0 kg/m^2^) and womenε[4.73 kg/m^2^, 5.7 kg/m^2^) (17), or handgrip strength menε[11.3 kg, 28 kg) and womenε[7.4 kg, 18 kg), (15) while severe thresholds were calf circumference men < 26.3cm and women < 25.2 cm or ASMI men < 5.68 kg/m^2^ and women < 4.73 kg/m^2^ or handgrip strength men < 11.3 kg and women < 7.4 kg.

#### Variables

Demographic characteristics in this study included: gender, age, and education level. Physical examinations included: height, weight, calf circumference and handgrip strength. Body composition measures included: appendicular skeletal muscle mass index (ASMI). Nutritional assessments included: Karnofsky performance status (KPS) score, reduced intake, and disease burden. The KPS were categorized into three levels: dependent level [0–40], semi-dependent level [50–70], and independent level [80–100]. The higher the KPS score, the better the health of the patient. Laboratory parameters included: white blood cell count (WBC), neutrophil percentage, lymphocyte percentage, red blood cell count (RBC), hemoglobin, platelet, glucose, blood urea nitrogen (BUN), creatinine, alanine aminotransferase (ALT), potassium, sodium, chloride, phosphorus, total protein and albumin. All of the above parameters were obtained within 24–48 h after patient admission.

### Outcomes

The primary hospitalization outcome measures of this study were length of stay and hospitalization costs, with the former defined as the number of days from admission to discharge and the latter including all treatment-related costs incurred during hospitalization.

### Quality control procedures

To ensure consistency, all interviewers and staff members received standardized pre-survey training. Initially, case report forms (CRFs) were completed in paper format and then entered into a secure electronic platform with logical checks by trained interviewers within 7 days. Quality control was implemented at multiple levels: local inspectors at each study site reviewed both paper and electronic CRFs, while a panel of experts randomly reviewed 10% of all CRFs via the electronic platform.

### Statistical analyses

In the data preprocessing stage, we addressed outliers in the measurement data. Values below the 1st percentile or above the 99th percentile for each variable were defined as outliers and replaced with random numbers between the 1st−25th percentile and the 75th–99th percentile, respectively. For missing data (Supplementary Table S1), mean imputation was used to fill in the missing values (20).

**Figure 1:**
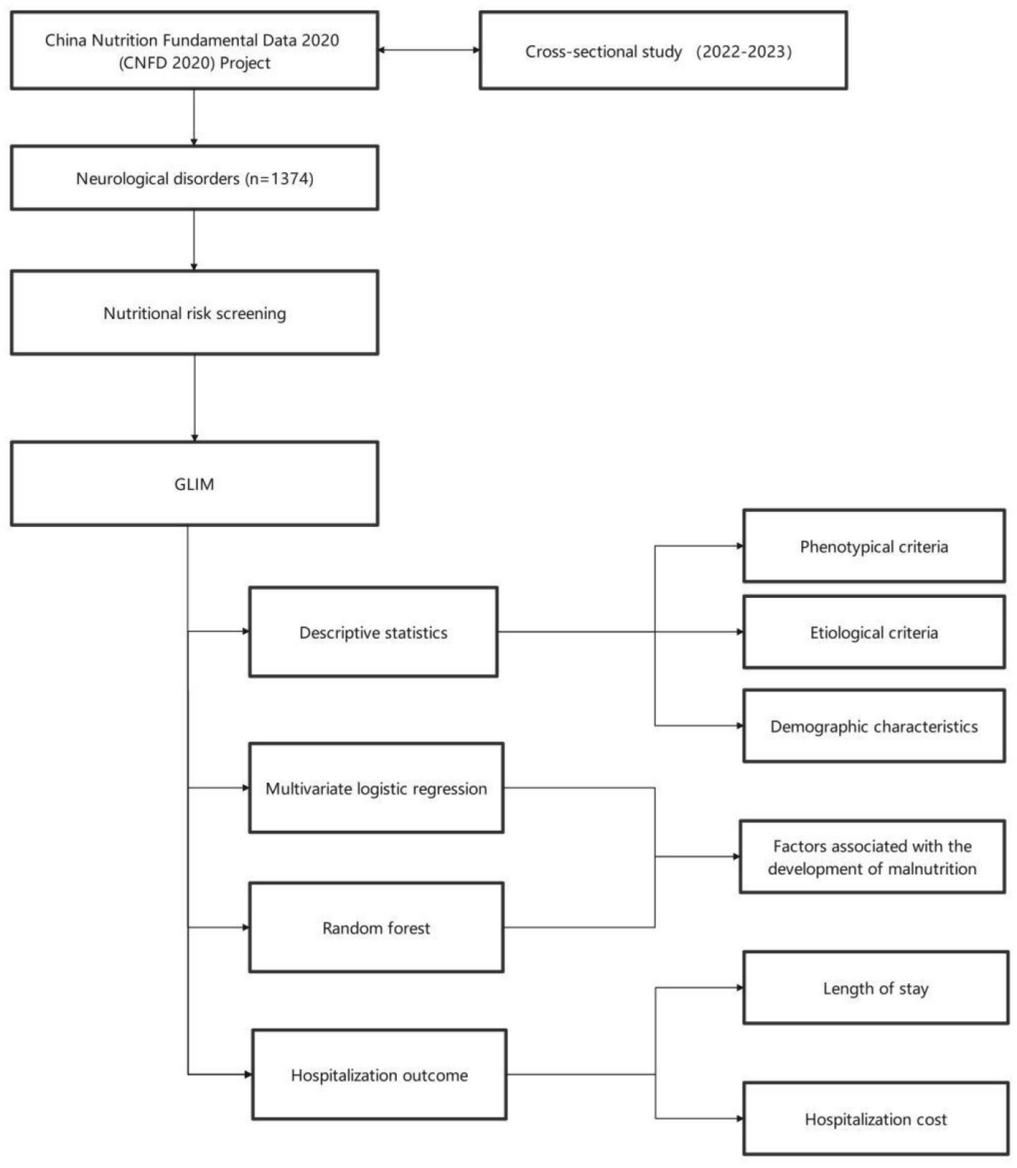
Flowchart of this study.

For quantitative data that did not meet the assumption of normal distribution, median and interquartile range (IQR) were used for descriptive statistics, and the Wilcoxon rank-sum test was used for comparisons between groups. Variables with statistical significance were screened by univariate analysis and included in a multivariate logistic regression. The specific variables are as follows: KPS, region, WBC, neutrophil percentage, lymphocyte percentage, RBC, hemoglobin, platelet, ALT, potassium, sodium, and chloride. Comparisons between groups were performed as follows: normal nutrition vs. malnutrition, normal nutrition vs. moderate malnutrition, normal nutrition vs. severe malnutrition, and moderate malnutrition vs. severe malnutrition, to analyze the factors influencing malnutrition. Results were presented as odds ratios (OR) with 95% confidence intervals (CI). Additionally, random forest analysis was performed using the same pairwise comparison approach as the logistic regression to model and analyze the predictive factors of malnutrition. In the random forest model, the data were divided into training and test sets in an 8:2 ratio, with synthetic minority oversampling technique (SMOTE) applied to the training set only. The parameters of the random forest model were set as follows: n_estimators =300, max_depth = 10, min_samples_split =10, min_samples_leaf = 4, max_features = “None,” and random_state = 42. The experiment was independently repeated 20 times, and the final results were averaged over the test sets. The final model interpretation was performed using SHapley Additive exPlanations (SHAP) theory to explain the predictions. The Kruskal-Wallis test and multiple linear regression were employed to analyze the associations between malnutrition status and hospitalization costs or length of stay, with results visualized using box plots. A threshold of *P* < 0.05 was set for statistical significance. All analyses were performed using R (version 4.4.3) and Python (version 3.11.4) (Figure 1).

## Results

### Demographic characteristics

This study included a total of 1,357 newly hospitalized patients initially diagnosed with neurological diseases from 31 provinces, autonomous regions, and municipalities directly under the central government in China. The results showed that the overall prevalence of malnutrition was 32.5%, with moderate malnutrition accounting for 30.8% and severe malnutrition accounting for 1.7%.

This study included 775 male patients and 582 female patients. The median age (IQR) was 64 (17.0) years. There were 11 patients with a Master's degree or higher, 174 patients with a Bachelor's degree, 293 patients with high school, 735 patients with primary or junior high school, and 144 patients with no formal education. There were 47 patients at dependent level, 294 patients at semi-dependent level, and 1,016 patients at independent level. There were 893 patients (65.8%) with comorbidities. The regional distribution of patients was as follows: 736 patients (54.2%) in the eastern region, 214 patients (15.8%) in the central region, and 407 patients (30.0%) in the western region. Of these patients, 441 were malnourished, with an overall malnutrition rate of 32.5%, including 418 patients with moderate malnutrition (30.8%) and 23 patients with severe malnutrition (1.7%) (Table 1). Laboratory characteristics and physical examination findings are shown in Supplementary Table S2.

**Table 1:** Demographic characteristics of neurological disorders.

**Characteristic**	**Total**	**Normal**	**Malnutrition**		
			**Total**	**Moderate**	**Severe**
Total, *n* (%)	1,357 (100.0)	916 (67.5)	441 (32.5)	386 (28.5)	55 (4.1)
**Age, years, median (IQR)**					
< 25	21.00 (4.0)	22.00 (4.0)	21.00 (3.5)	21.00 (3.5)	19.00 (0.0)
25-35	31.00 (5.0)	30.00 (5.0)	32.00 (3.0)	32.00 (5.0)	32.50 (0.5)
35-45	40.00 (6.0)	40.00 (6.0)	41.00 (3.5)	41.00 (4.0)	41.00 (5.0)
45-55	50.00 (5.0)	50.00 (5.0)	50.50 (6.8)	51.00 (7.0)	48.50 (5.5)
55-65	59.00 (5.0)	59.00 (4.3)	60.00 (5.0)	60.00 (5.0)	60.00 (5.0)
65-75	69.00 (5.0)	69.00 (4.0)	70.00 (4.0)	70.00 (4.3)	70.00 (4.0)
75-85	78.00 (5.0)	78.00 (5.0)	78.00 (5.0)	78.00 (5.0)	78.50 (4.8)
≥85	86.00 (4.0)	86.00 (3.0)	86.50 (5.3)	86.00 (4.0)	90.00 (3.5)
**Gender**, ***n*** **(%)**					
Male	775 (57.1)	531 (58.0)	244 (55.3)	217 (56.2)	27 (49.1)
Female	582 (42.9)	385 (42.0)	197 (44.7)	169 (43.8)	28 (50.9)
**Education level**, ***n*** **(%)**					
Master's degree or higher	11 (0.8)	9 (1.0)	2 (0.5)	2 (0.5)	0 (0.0)
Bachelor's degree	174 (12.8)	121 (13.2)	53 (12.0)	46 (11.9)	7 (12.7)
High school	293 (21.6)	220 (24.0)	73 (16.6)	67 (17.4)	6 (10.9)
Primary or junior high school	735 (54.2)	478 (52.2)	257 (58.3)	226 (58.5)	31 (56.4)
No formal education	144 (10.6)	88 (9.6)	56 (12.7)	45 (11.7)	11 (20.0)
**KPS**, ***n*** **(%)**					
Dependent level	47 (3.5)	18 (2.0)	29 (6.6)	18 (4.7)	11 (20.0)
Semi-dependent level	294 (21.7)	147 (16.0)	147 (33.3)	130 (33.7)	17 (30.9)
Independent level	1,016 (74.9)	751 (82.0)	265 (60.1)	238 (61.7)	27 (49.1)
**Comorbidity**, ***n*** **(%)**					
Yes	893 (65.8)	578 (63.1)	315 (71.4)	279 (72.3)	36 (65.5)
No	464 (34.2)	338 (36.9)	126 (28.6)	107 (27.7)	19 (34.5)
**Region**, ***n*** **(%)**					
Eastern	736 (54.2)	504 (55.0)	232 (52.6)	204 (52.8)	28 (50.9)
Central	214 (15.8)	163 (17.8)	51 (11.6)	46 (11.9)	5 (9.1)
Western	407 (30.0)	249 (27.2)	158 (35.8)	136 (35.2)	22 (40.0)

The eastern region had a significantly higher proportion of normal patients (55.0%) compared to its malnourished patients (52.6%), while the central region had 163 (17.8%) normal patients compared to 51 (11.6%) malnourished patients. In contrast, the western region demonstrated a significantly higher prevalence of malnutrition (35.8%) compared to its normal population (27.1%). Univariate analysis confirmed a significant association between regional differences and nutritional status (*P* = 0.042), suggesting that the western region may be a risk area for malnutrition (Table 1, Supplementary Table S2).

### Status of malnutrition in neurological disorders

Phenotypic results findings: 428 patients (31.5%) with moderate malnutrition had weight loss, 13 patients (1.0%) with severe malnutrition had weight loss; 417 patients (30.7%) with moderate malnutrition had low BMI, 24 patients (1.8%) with severe malnutrition had a low BMI; 412 patients (30.4%) with moderate malnutrition had muscle loss, and 29 patients (2.1%) with severe malnutrition had muscle loss. Compared with other neurological disorders, patients with cerebrovascular disease were the most likely to meet the phenotypic criteria (59.1%). The western region (1.2%, 2.0%, 4.2%) exhibited the highest prevalence of the three malnutrition phenotypes compared to the eastern (1%, 1.8%, 1.5%) and central (0.5%, 1.4%, 0.5%) regions (Table 2).

**Table 2:** GLIM phenotypic criteria for neurological disorders.

**Group**	**Total, *n* (%)**	**Phenotypical criteria**, ***n*** **(%)**								
		**Weight loss**			**Low BMI**			**Reduced muscle mass**		
		**Normal**	**Moderate**	**Severe**	**Normal**	**Moderate**	**Severe**	**Normal**	**Moderate**	**Severe**
Total	1,357 (100.0)	916 (67.5)	428 (31.5)	13 (1.0)	916 (67.5)	417 (30.7)	24 (1.8)	916 (67.5)	412 (30.4)	29 (2.1)
**Types of diseases**										
Cerebrovascular disease	802 (59.1)	518 (64.6)	277 (34.5)	7 (0.9)	518 (64.6)	270 (33.7)	14 (1.8)	518 (64.6)	264 (32.9)	20 (2.5)
Dizziness	123 (9.1)	93 (75.6)	29 (23.6)	1 (0.8)	93 (75.6)	28 (22.8)	2 (1.6)	93 (75.6)	29 (23.6)	1 (0.8)
Peripheral nerve disorders	122 (9.0)	92 (75.4)	29 (23.8)	1 (0.8)	92 (75.4)	29 (23.8)	1 (0.8)	92 (75.4)	30 (24.6)	0 (0.0)
Central nervous system disorders	115 (8.5)	72 (62.6)	41 (35.7)	2 (1.7)	72 (62.6)	40 (34.8)	3 (2.6)	72 (62.6)	40 (34.8)	3 (2.6)
Headache	36 (2.7)	30 (83.3)	5 (13.9)	1 (2.8)	30 (83.3)	5 (13.9)	1 (2.8)	30 (83.3)	6 (16.7)	0 (0.0)
Movement disorders	36 (2.7)	23 (63.9)	12 (33.3)	1 (2.8)	23 (63.9)	12 (33.3)	1 (2.8)	23 (63.9)	11 (30.6)	2 (5.6)
Epilepsy	21 (1.6)	13 (61.9)	8 (38.1)	0 (0.0)	13 (61.9)	7 (33.3)	1 (4.8)	13 (61.9)	6 (28.6)	2 (9.5)
Spinal cord disorders	10 (0.7)	8 (80.0)	2 (20.0)	0 (0.0)	7 (70.0)	3 (30.0)	0 (0.0)	8 (80.0)	2 (20.0)	0 (0.0)
Neuromuscular muscle diseases	10 (0.7)	6 (60.0)	4 (40.0)	0 (0.0)	6 (60.0)	4 (40.0)	0 (0.0)	6 (60.0)	3 (30.0)	1 (10.0)
Mental disorders	13 (1.0)	7 (53.9)	6 (46.2)	0 (0.0)	7 (53.9)	6 (46.2)	0 (0.0)	7 (53.9)	6 (46.2)	0 (0.0)
Others	69 (5.1)	54 (78.3)	15 (21.7)	0 (0.0)	54 (78.3)	14 (20.3)	1 (1.5)	54 (78.3)	15 (21.8)	0 (0.0)
**Region**										
Eastern	736 (54.2)	504 (68.5)	225 (30.6)	7 (1.0)	504 (68.5)	219 (29.8)	13 (1.8)	504 (68.5)	221 (30.0)	11 (1.5)
Central	214 (15.8)	163 (76.2)	50 (23.4)	1 (0.5)	163 (76.2)	48 (22.4)	3 (1.4)	163 (76.2)	50 (23.4)	1 (0.5)
Western	407 (30.0)	249 (61.2)	153 (37.6)	5 (1.2)	249 (61.2)	150 (36.9)	8 (2.0)	249 (61.2)	141 (34.6)	17 (4.2)

Etiologic results findings: 342 patients (25.2%) with malnutrition showed reduced food intake/assimilation, and 423 patients (31.7%) with malnutrition showed disease burden/inflammation. The western region (35.6%, 32.4%) showed the highest prevalence of malnutrition meeting both etiologic criteria compared to the central (14.0%, 28.0%) and eastern (22.7%, 31.4%) regions (Table 3).

**Table 3:** GLIM etiological criteria for neurological disorders.

**Variable**	**Total, *n* (%)**	**Etiological criteria**, ***n*** **(%)**			
		**Reduced food intake or assimilation**		**Disease burden/inflammation**	
		**Normal**	**Malnutrition**	**Normal**	**Malnutrition**
Total	1,357 (100.0)	1,015 (74.8)	342 (25.2)	934 (68.8)	423 (31.2)
**Types of diseases**			
Cerebrovascular disease	802 (59.1)	590 (73.6)	212 (26.4)	548 (68.3)	254 (31.7)
Dizziness	123 (9.1)	102 (82.9)	21 (17.1)	97 (78.9)	26 (21.1)
Peripheral nerve disorders	122 (9.0)	97 (79.5)	25 (20.5)	84 (68.9)	38 (31.2)
Central nervous system disorders	115 (8.5)	78 (67.8)	37 (32.2)	68 (59.1)	47 (40.9)
Headache	36 (2.7)	31 (86.2)	5 (13.9)	28 (77.8)	8 (22.2)
Movement disorders	36 (2.7)	25 (69.4)	11 (30.6)	25 (69.4)	11 (30.6)
Epilepsy	21 (1.6)	17 (81.0)	4 (19.1)	9 (42.9)	12 (57.1)
Spinal cord disorders	10 (0.7)	8 (80.0)	2 (20.0)	7 (70.0)	3 (30.0)
Neuromuscular muscle diseases	10 (0.7)	6 (60.0)	4 (40.0)	4 (40.0)	6 (60.0)
Mental disorders	13 (1.0)	7 (53.9)	6 (46.2)	10 (76.9)	3 (23.1)
Others	69 (5.1)	54 (78.3)	15 (21.7)	54 (78.3)	15 (21.7)
**Region**					
Eastern	736 (54.2)	569 (77.3)	167 (22.7)	505 (68.6)	231 (31.4)
Central	214 (15.8)	184 (86.0)	30 (14.0)	154 (72.0)	60 (28.0)
Western	407 (30.0)	262 (64.4)	145 (35.6)	275 (67.6)	132 (32.4)

### Multivariable logistic regression

Based on the results of the univariate analysis (Supplementary Table S3), we excluded non-significant factors (*P* > 0.05) and entered the remaining variables into logistic regression models, followed by stepwise multivariate logistic regression analyses across different nutritional status groups. In the malnutrition vs. normal group, the higher the KPS score the lower the risk of malnutrition (*P* < 0.05). The risk of malnutrition was lower in the central region (*P* = 0.015) and higher in the western region (*P* = 0.048) compared to the eastern region. The risk of malnutrition was higher with higher neutrophil percentage, lower RBC and chloride (*P* < 0.05). In moderate malnutrition vs. normal group, KPS score, region, neutrophil percentage and RBC were similar to that of malnutrition vs. normal group. The risk of malnutrition was higher with less chloride (*P* = 0.011). ALT had no statistical significance. In the severe malnutrition vs. normal group, KPS score, neutrophil percentage and chloride were similar to those in the moderate malnutrition vs. normal group. Lower hemoglobin, platelets and potassium were associated with a higher risk of malnutrition (*P* < 0.05). In severe vs. moderate malnutrition, KPS score, chloride, hemoglobin and potassium were similar to those in severe malnutrition vs. normal group. Platelets were not statistically significant (Table 4).

**Table 4:** Multivariate logistic regression.

**Variables**	**OR**	**Upper limit**	**Lower limit**	***P***
**Normal vs. Malnutrition**				
KPS, Dependent level (Ref)				
Semi-dependent level	0.923	0.469	1.785	0.814
Independent level	0.381	0.198	0.721	0.003
Region, Eastern region (Ref)				
Central region	0.632	0.433	0.910	0.015
Western region	1.319	1.002	1.734	0.048
Neutrophil percentage (%)	1.026	1.015	1.038	< 0.001
RBC (10^12^/L)	0.528	0.425	0.655	< 0.001
Chloride (mmol/L)	0.947	0.915	0.979	0.002
**Normal vs. Moderate malnutrition**				
KPS, Dependent level (Ref)				
Semi-dependent level	1.184	0.572	2.453	0.648
Independent level	0.482	0.238	0.979	0.042
Region, Eastern region (Ref)				
Central region	0.652	0.441	0.950	0.029
Western region	1.282	0.964	1.702	0.087
Neutrophil percentage (%)	1.025	1.013	1.037	< 0.001
RBC (10^12^/L)	0.572	0.456	0.716	< 0.001
ALT (U/L)	0.994	0.987	1.002	0.141
Chloride (mmol/L)	0.955	0.921	0.989	0.011
**Normal vs. Severe malnutrition**				
KPS, Dependent level (Ref)				
Semi-dependent level	0.337	0.118	0.985	0.043
Independent level	0.145	0.055	0.398	< 0.001
Neutrophil percentage (%)	1.049	1.022	1.077	< 0.001
Hemoglobin (g/L)	0.960	0.943	0.977	< 0.001
Platelet (10^9^/L)	0.995	0.990	1.000	0.040
Potassium (mmol/L)	0.579	0.373	0.906	0.016
Chloride (mmol/L)	0.901	0.841	0.968	0.003
**Moderate vs. Severe malnutrition**				
KPS, Dependent level (Ref)				
Semi-dependent level	0.261	0.100	0.688	0.006
Independent level	0.287	0.117	0.732	0.007
Hemoglobin (g/L)	0.982	0.966	0.998	0.033
Platelet (10^9^/L)	0.996	0.992	1.001	0.139
Potassium (mmol/L)	0.593	0.380	0.923	0.020
Chloride (mmol/L)	0.916	0.853	0.986	0.017

KPS, Karnofsky performance status; RBC, red blood cell count; ALT, alanine aminotransferase.

### Random forest

**Figure 2:**
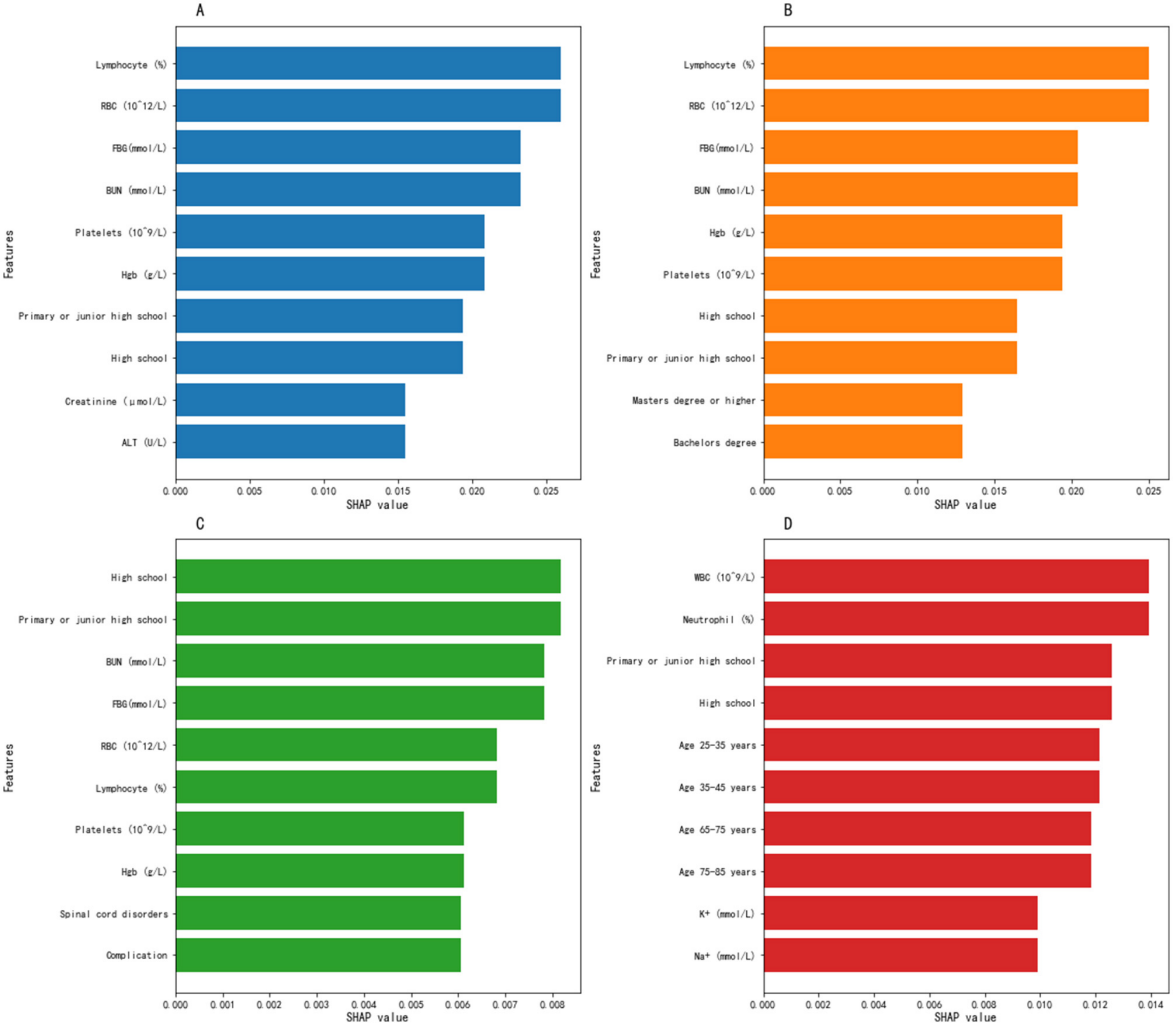
SHAP plots of the top 10 predictors of malnutrition. **(A)** Normal vs. malnutrition groups; **(B)** normal vs. moderate malnutrition groups; **(C)** normal vs. severe malnutrition groups; **(D)** moderate vs. severe malnutrition groups.

Figure 2 displays the top ten most important predictive factors for comparisons between the four malnutrition groups. Figure 2A presents the top 10 predictors for malnutrition, with lymphocyte percentage, RBC, and glucose ranking in the top three, while the top five factors include lymphocyte percentage, RBC, glucose, BUN, and platelet count. Figure 2B shows the top 10 predictors of moderate malnutrition, with lymphocyte percentage, RBC, and glucose as the top three, and the top five being lymphocyte percentage, RBC, glucose, BUN, and hemoglobin. Figure 2C illustrates the top 10 predictors of severe malnutrition, with senior high school education, primary/junior high school education, and BUN ranking highest, followed by senior high school education, primary/junior high school education, BUN, glucose, and RBC in the top five. Figure 2D demonstrates the top 10 factors predicting severe malnutrition among moderate malnutrition patients, with WBC, neutrophil percentage, and primary/junior high school education level being the top three predictors, while the top five consist of WBC, neutrophil percentage, primary/junior high school education, senior high school education, and age 25–35 years.

**Figure 3:**
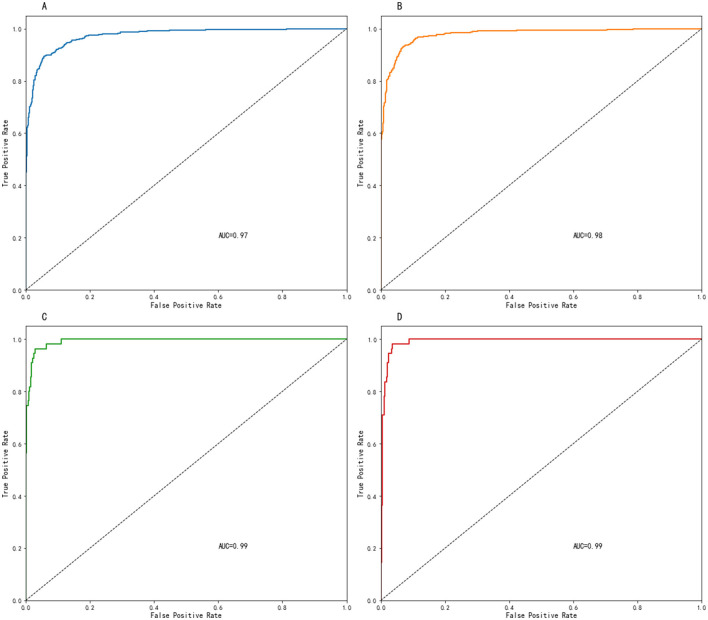
ROC curves. **(A)** Normal vs. malnutrition groups; **(B)** normal vs. moderate malnutrition groups; **(C)** normal vs. severe malnutrition groups; **(D)** moderate malnutrition vs. severe malnutrition groups.

The developed random forest prediction models performed comparisons between the three malnutrition classification groups and the normal nutrition group, with all models demonstrating excellent predictive performance. Specifically, the models corresponding to Figures 3C, D achieved the highest AUC values of 0.99, followed by Figure 3B (AUC = 0.97), and Figure 3A (AUC = 0.97). Full model performance metrics, including accuracy rates and detailed AUC values for all four models, are provided in Supplementary Table S4.

### Clinical impact

**Figure 4:**
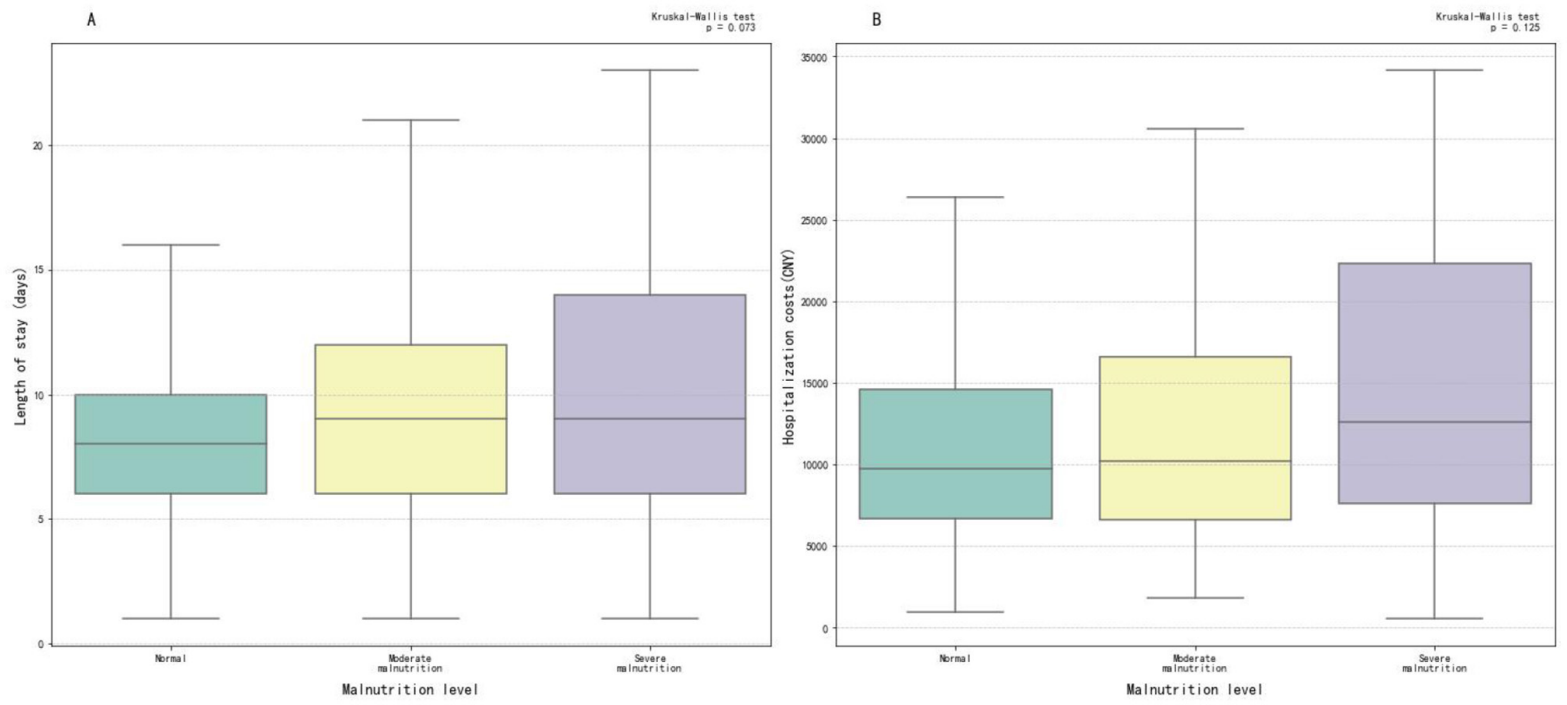
Box plots of length of stay and hospitalization costs. **(A)** Malnutrition and length of stay group; **(B)** malnutrition and hospitalization costs group.

Figure 4 presents boxplot analyses of the relationship between patient malnutrition status and hospitalization outcomes. As shown in Figure 4A, the median length of stay increased progressively with worsening malnutrition severity, with malnourished patients having longer length of stay than the normal nutrition group, along with more dispersed data distributions and larger IQRs. Figure 4B shows that hospitalization costs increased with the severity of malnutrition. Patients with severe malnutrition had higher hospitalization costs than those with normal nutrition, with costs slightly exceeding those of moderately malnourished patients. Both the Kruskal-Wallis test and multiple linear regression showed no statistically significant difference in malnutrition. OR and 95% CI are shown in Supplementary Table S5.

## Discussion

In this study the main findings are as follows: First, we found a high overall prevalence of malnutrition (32.5%), with moderate malnutrition being far more common (30.8%) than severe malnutrition (1.7%). Second, neutrophil percentage, lymphocyte percentage, red blood cell count, western region and education level were identified as influencing factors. Third, malnutrition showed a trend toward being associated with increased length of hospital stay and higher hospitalization costs.

Current research on malnutrition in neurological disorders primarily focuses on specific disease subtypes (e.g., Parkinson's disease, acute ischemic stroke) or particular populations (e.g., elderly patients), and most studies being single-center investigations (11, 12). Furthermore, the majority of existing studies have either analyzed only the influencing factors of malnutrition or examined solely the relationship between malnutrition and clinical outcomes (21, 22). Existing literature indicates that the GLIM criteria have been applied to the diagnosis of nutritional status in patients with gastrointestinal, cardiovascular, and oncological diseases, whereas rarely in neurological disorders (23–25). This study utilized data covering 31 provinces, municipalities, and autonomous regions in China, with a sample size of 1,357 patients. It is one of the few studies with nationwide coverage and is the first multicentre cross-sectional study to systematically report the distribution of GLIM indicators in patients with neurological disorders in China. It is also one of the few studies to comprehensively elucidate the current status of malnutrition in neurological diseases. The identification of associated factors and their impact on hospitalization outcomes has significant implications for improving patient prognosis.

### GLIM criteria

By comparing the distribution of GLIM indicators across different neurological disease types and regions in China, we found that the western region had significantly higher proportions of severe phenotypic malnutrition indicators and etiologic malnutrition indicators than eastern and central regions. We hypothesize that this disparity may be due to developmental differences in education, economy, and health infrastructure in western regions compared with eastern/central regions, which may exacerbate the severity of malnutrition (2, 25). Furthermore, patients with cerebrovascular disease experienced higher rates of moderate weight loss and low BMI than those with other neurological conditions. This may be due to hypermetabolic states (e.g., spasticity) and chronic inflammation (2, 7). Patients with central nervous system disorders exhibited particularly pronounced moderate to severe muscle mass reduction, suggesting that neurogenic muscle atrophy may represent the core phenotypic manifestation of malnutrition in these disorders.

### Neurological mechanisms of malnutrition

The high prevalence of malnutrition in neurological patients may be attributed to impaired nutrient intake and disease-specific mechanisms. For instance, patients with stroke or Parkinson's disease often experience loss of arm strength and coordination, leading to difficulties in self-feeding (26, 27). Those with Alzheimer's disease or other dementias may forget to eat due to cognitive decline (28). Additionally, financial constraints and mobility limitations—common in chronic neurological conditions—can further restrict access to adequate nutrition. The use of the KPS helps quantify functional and cognitive impairments, while demographic variables such as education level and geographic region serve as proxies for socioeconomic and environmental barriers that affect both disease management and nutritional access. By integrating functional metrics like the KPS with demographic factors, our study captures this relationship.

### Prevalence of malnutrition

Chen et al. used GLIM to investigate the association between malnutrition and neurological disease in elderly patients, with a sample size of 566 patients. Their results showed a malnutrition rate of 14.7%, indicating certain associations between malnutrition and several hospitalization indicators/outcomes (29). Liu et al. (1) applied both GLIM and subjective global assessment (SGA) to investigate the relationship between malnutrition and clinical outcomes in neurocritical patients. This single-center study (*n* = 161) reported malnutrition rates of 49% (GLIM) and 28% (SGA). The study showed that 22% of malnourished patients met GLIM criteria while only 1% met SGA criteria, demonstrating the superiority of GLIM in diagnosing malnutrition in neurological patients. The single-center study by Zhang et al. (30) (*n* = 140) used NRS-2002, nutrition risk in the critically ill (NUTRIC) and modified NUTRIC (mNUTRIC) to assess malnutrition in critically ill neurological patients and found nutrition risk rates of 87.1%, 15.7% and 28.6%, respectively. Our study found a malnutrition rate of 32.5% in neurological patients (moderate: 30.8%; severe: 1.7%). We attribute this increased prevalence primarily to disease-specific factors such as dysphagia and reduced food intake due to stroke, dementia and other neurological conditions.

As a multicentre study covering 31 provinces with 1,357 patients using the GLIM criteria, our methodology differed significantly from Liru et al.'s (29) study which used only calf circumference (male ≤ 30 cm, female ≤ 29 cm) to assess muscle mass. We implemented three internationally recognized measures: calf circumference, ASMI, and handgrip strength. These methodological differences in nutritional assessment criteria and indicators are likely to account for the substantial variation in reported malnutrition rates.

### Factors influencing malnutrition

Using logistic regression and random forest analyses, we identified neutrophil percentage, lymphocyte percentage, western region, and RBC as significant influencing factors for malnutrition. The study by Kaya et al. demonstrated that the neutrophil-to-lymphocyte ratio (NLR) was an independent risk factor for malnutrition in elderly patients (3). As an inflammatory marker, the NLR reflects the systemic inflammatory status - elevated NLR values (indicating increased neutrophils and decreased lymphocytes) indicate impaired inflammatory homeostasis. Our findings regarding neutrophil and lymphocyte percentages as determinants of malnutrition are consistent with these previous reports. In addition, lower RBC levels indicate a higher likelihood of anemia and an increased risk of malnutrition. Research by Göl et al. (31) suggests a correlation between malnutrition and anemia, which is consistent with the findings of the present study. The consistent prominence of primary/junior high school education level among the top-ranked determinants of different malnutrition statuses suggests that lower educational attainment may impair patients' ability to manage their nutritional needs. Lower educational attainment is often associated with limited financial resources, which may restrict access to adequate food and healthcare services. These socioeconomic barriers can manifest as insufficient nutrient intake and delayed medical intervention, thereby collectively increasing the risk of malnutrition.

In the multivariate logistic regression, the WBC was not statistically significant, but it ranked first in terms of contribution in the random forest model (Figure 2D). We believe the possible reason for this is that multivariate logistic regression calculates regression coefficients based on linear assumptions, placing more emphasis on interpretability and highlighting linear relationships, whereas random forests assess feature contributions to predictions, prioritizing predictive performance and providing some ability to identify non-linear relationships and interactions. Additionally, the former determines significance, quantifies actual impact, and interprets key factors by incorporating confidence intervals, whereas the latter first confirms whether importance is stable, validates it with domain knowledge, and finally quantifies the actual impact to explain key factors. As a result, the WBC produces different results depending on the algorithm used, but it still has some indicative value.

### Impact of malnutrition on hospitalization outcomes

Numerous studies have studied the relationship between malnutrition in patients with neurological disease and short-term clinical outcomes. Li et al. (7) investigated stroke-associated pneumonia (SAP) using logistic regression models. They demonstrated that malnutrition was common in ischemic stroke patients and was associated with an increased risk of stroke-associated pneumonia, suggesting that early nutritional assessment tools could help identify high-risk SAP patients. Cai et al.'s logistic regression analysis showed that the risk of malnutrition correlated with poor outcomes at three months (15, 21). Our study employed Kruskal-Wallis test and multiple linear regression to analyze impact of malnutrition on the costs and duration of hospitalization. The results showed progressively increasing costs as the severity of malnutrition worsened, probably due to greater use of medical resources for recovery. Longer length of stay in malnourished patients may reflect extended rehabilitation needs. Furthermore, the study by Ruiz et al. (32) showed that malnutrition leaded to increased hospitalization costs, while Correia and Waitzberg identified malnutrition as an independent risk factor for prolonged hospital stays and higher medical costs, findings that are consistent with our results (2).

However, the current study has limitations. We did not assess the representativeness of the sample or use a national sample with fully randomized data. The determination of weight loss was based on patients' self-reported recall at the six-month mark, which introduces subjective bias into the assessment of malnutrition. To maximize the accuracy of this data, we excluded patients with psychiatric or memory disorders; however, this approach may have affected the enrollment of conditions such as Parkinson's disease and Alzheimer's disease. Lack of data on National Institute of Health Stroke Scale (NIHSS) scores, comorbidities, and medication use prevented a complete analysis of the impact of confounders on malnutrition and hospitalization outcomes. Due to the cross-sectional nature of this study, the causal relationship between the influencing factors and malnutrition could not be tested. Future research should assess sample representativeness, reduce subjectivity in weight reporting, compare different assessment methods for national assessment of malnutrition in neurological disease, and conduct prospective studies to further investigate these causal relationships.

In conclusion, our study reveals the malnutrition status and GLIM indicator distribution of neurological disease in China. The results demonstrate the high prevalence of malnutrition in these patients, with neutrophil percentage, lymphocytes, RBC, western region, and education level as influencing factors. Malnutrition may increase the length of stay and hospitalization costs. Given its high incidence and impact on healthcare resources, clinical practice should prioritize assessment of nutritional status on admission. Assessments should take full account of laboratory indicators and individual variability, recognizing differences between methods. The GLIM criteria adopted here provide a more comprehensive assessment of malnutrition.

## Data Availability

The data analyzed in this study is subject to the following licenses/restrictions: The study data that underlie the results of this article will be available for investigators after approval by the National Institute of Hospital Administration (Beijing, China). Please email the corresponding author for more information. Requests to access these datasets should be directed to Siping Dong, sipingd@163.com.
