# Supplementary material for: Status quo, influencing factors, and association with hospitalization outcomes of malnutrition in neurological disorders in China: a national cross-sectional study
Source: Front Nutr. 2025 Nov 7;12:1633212. doi: 10.3389/fnut.2025.1633212 (PMC12635621; doi:10.3389/fnut.2025.1633212)
Supplement: Supplementary file 1 [file Table_1.docx]

# **Supplementary Materials**

# **Status quo, influencing factors, and association with hospitalization outcomes of malnutrition in neurological disorders in China: a national cross-sectional study**

**Table S1.** Missing data.

**Table S2.** Laboratory and physical examination of neurological disorders.

**Table S3.** Comparison between normal and malnutrition groups.

**Table S4.** Classification performance with random forest.

**Table S5.** Association between malnutrition and hospitalization outcomes.

**Table S1. Missing data**

| Laboratory | Missing rate |
| --- | --- |
| WBC (109/L) | 2.2% |
| Neutrophil percentage (%) | 2.2% |
| Lymphocyte percentage (%) | 2.3% |
| RBC (1012/L) | 2.2% |
| Hemoglobin (g/L) | 2.2% |
| Platelet (109/L) | 2.2% |
| glucose (mmol/L) | 8.1% |
| BUN (mmol/L) | 5.6% |
| Creatinine (μmol/L) | 4.1% |
| ALT (U/L) | 4.1% |
| Potassium (mmol/L) | 2.7% |
| Sodium (mmol/L) | 2.8% |
| Chloride(mmol/L) | 2.9% |
| Phosphorus (mmol/L) | 23.6% |
| Total protein (g/L) | 5.0% |
| Albumin (g/L) | 4.1% |

WBC: white blood cell count; RBC: red blood cell count; ALT: alanine aminotransferase; BUN: blood urea nitrogen

**Table S2. Laboratory and physical examination of neurological disorders.**

| Characteristic | Total | Normal | Malnutrition | | |
| --- | --- | --- | --- | --- | --- |
|  |  |  | Total | Moderate | Severe |
| Laboratory ,median(IQR) |  |  |  |  |  |
| WBC (10^9^/L) | 6.20 (2.51) | 6.18 (2.46) | 6.21 (2.69) | 6.15 (2.50) | 6.57 (4.05) |
| Neutrophils percentage (%) | 62.50 (14.45) | 61.40 (13.55) | 65.50 (16.50) | 65.45 (15.83) | 66.30 (20.40) |
| Lymphocyte percentage (%) | 27.14 (12.85) | 27.95 (11.88) | 24.90 (14.10) | 25.10 (14.00) | 22.30 (18.70) |
| RBC (10^12^/L) | 4.45 (0.72) | 4.51 (0.69) | 4.35 (0.76) | 4.38 (0.74) | 4.19 (0.79) |
| Hemoglobin (g/L) | 136.00 (21.00) | 137.00 (21.00) | 133.00 (21.00) | 133.00 (21.00) | 126.00 (23.00) |
| Platelet (10^9^/L) | 210.00 (79.00) | 210.50 (75.00) | 208.00 (85.50) | 210.00 (83.25) | 196.00 (105.00) |
| Glucose (mmol/L) | 5.67 (1.87) | 5.58 (1.77) | 5.75 (2.06) | 5.74 (2.05) | 5.78 (2.17) |
| BUN (mmol/L) | 5.27 (2.18) | 5.30 (1.99) | 5.20 (2.53) | 5.19 (2.37) | 5.53 (4.12) |
| Creatinine (μmol/L) | 68.00 (24.30) | 68.65 (24.10) | 67.00 (26.65) | 67.60 (26.00) | 66.00 (27.30) |
| ALT (U/L) | 18.60 (14.00) | 19.00 (13.70) | 17.00 (13.30) | 17.00 (13.00) | 16.00 (17.80) |
| Potassium (mmol/L) | 3.86 (0.49) | 3.88 (0.49) | 3.83 (0.53) | 3.86 (0.52) | 3.69 (0.53) |
| Sodium (mmol/L) | 140.00 (3.75) | 140.15 (3.30) | 140.00 (3.65) | 140.00 (3.70) | 139.00 (4.10) |
| Chloride (mmol/L) | 105.30 (4.00) | 105.70 (3.58) | 105.00 (4.65) | 105.01 (4.40) | 103.30 (5.90) |
| Phosphorus (mmol/L) | 1.08 (0.19) | 1.08 (0.16) | 1.08 (0.20) | 1.08 (0.20) | 1.08 (0.23) |
| Total protein (g/L) | 66.60 (7.60) | 66.60 (7.48) | 66.00 (8.20) | 66.05 (8.00) | 65.00 (11.20) |
| Albumin (g/L) | 39.90 (5.00) | 40.00 (4.90) | 39.70 (5.60) | 39.76 (5.30) | 39.10 (8.30) |
| Physical examination,median(IQR) | |  |  |  |  |
| Height (cm) | 165.00 (12.00) | 165.00 (11.00) | 163.00 (12.00) | 163.30 (12.00) | 163.00 (12.00) |
| Weight (kg) | 65.00 (18.00) | 67.00 (16.20) | 60.50 (17.00) | 61.00 (16.00) | 55.00 (33.10) |
| Calf circumference (cm) | 34.00 (5.40) | 34.60 (5.00) | 32.00 (5.00) | 32.00 (4.80) | 33.00 (8.70) |
| Handgrip strength (kg) | 23.40 (16.30) | 26.30 (16.40) | 18.00 (13.20) | 19.20 (11.85) | 7.10 (11.80) |
| ASMI (kg/m^2^) | 7.72 (2.61) | 7.89 (2.60) | 7.27 (2.53) | 7.38 (2.52) | 6.29 (2.17) |

KPS: Karnofsky performance status; WBC: white blood cell count; RBC: red blood cell count; ALT: alanine aminotransferase; BUN: blood urea nitrogen; ASMI: appendicular skeletal muscle mass index.

**Table S3. Comparison between normal and malnutrition groups**.

| Variable | *P* |
| --- | --- |
| Age | 0.547 |
| Gender | 0.375 |
| Eeducation level | 0.341 |
| KPS | <0.001 |
| Comorbidity | 0.220 |
| Types of Diseases | 0.125 |
| Region | 0.042 |
| **Laboratory** |  |
| WBC (10^9^/L) | 0.075 |
| Neutrophil percentage (%) | <0.001 |
| Lymphocyte percentage (%) | <0.001 |
| RBC (10^12^/L) | 0.004 |
| Hemoglobin (g/L) | 0.005 |
| Platelet (10^9^/L) | 0.012 |
| glucose (mmol/L) | 0.319 |
| BUN (mmol/L) | 0.430 |
| Creatinine (μmol/L) | 0.108 |
| ALT (U/L) | 0.026 |
| Potassium (mmol/L) | 0.005 |
| Sodium (mmol/L) | 0.033 |
| Chloride(mmol/L) | 0.002 |
| Phosphorus (mmol/L) | 0.814 |
| Total protein (g/L) | 0.208 |
| Albumin (g/L) | 0.526 |

KPS: Karnofsky performance status; WBC: white blood cell count; RBC: red blood cell count; ALT: alanine aminotransferase; BUN: blood urea nitrogen

**Table S4.** **Classification performance with random forest**.

| Group | Accuracy | AUC |
| --- | --- | --- |
| Normal vs Malnutrition | 85.777% | 0.972 |
| Normal vs Moderate malnutrition | 85.330% | 0.977 |
| Normal vs Severe malnutrition | 94.953% | 0.993 |
| Moderate malnutrition vs Severe malnutrition | 88.889% | 0.990 |

**Table S5. Association between malnutrition and hospitalization outcomes**.

| Comparison | OR | Upper limit | Lower limit |
| --- | --- | --- | --- |
| Length of stay | | | |
| Normal vs Moderate malnutrition | 1.034 | 1.059 | 1.009 |
| Normal vs Severe malnutrition | 1.057 | 1.107 | 1.009 |
| Moderate malnutrition vs Severe malnutrition | 1.025 | 1.066 | 0.972 |
| Hospitalization costs | | | |
| Normal vs Moderate malnutrition | 1.000 | 1.000 | 1.000 |
| Normal vs Severe malnutrition | 1.000 | 1.000 | 1.000 |
| Moderate malnutrition vs Severe malnutrition | 1.000 | 1.000 | 1.000 |
